# Preoperative psychological distress no reason to delay total knee arthroplasty: a register-based prospective cohort study of 458 patients

**DOI:** 10.1007/s00402-020-03537-w

**Published:** 2020-07-28

**Authors:** Aamir Mahdi, Maria Hälleberg-Nyman, Per Wretenberg

**Affiliations:** 1 Department of Orthopaedics, Örebro County Council, Örebro, Sweden; 2 School of Medical Sciences, Örebro University, Örebro, Sweden; 3 School of Health Sciences, Örebro University, Örebro, Sweden; 4 Department of Orthopaedics, Faculty of Health and Medicine, Örebro University, SE-701 82 Örebro, Sweden

**Keywords:** Knee arthritis, Knee Injury and Osteoarthritis Outcome Score, Psychological distress, Total knee arthroplasty

## Abstract

**Introduction:**

Total knee arthroplasty (TKA) is effective in alleviating pain and improving function in patients with knee arthritis. Psychological factors are known to affect patient satisfaction after TKA. It is important to determine the effectiveness of TKA in patients with presurgical anxiety and/or depression to avoid excluding them from surgery.

**Materials and methods:**

A prospective cohort study was conducted on all patients who underwent TKA during 2016–2018. Patients were divided into four groups: with anxiety, without anxiety, with depression, and without depression. Outcome measures comprised both generic and knee-specific instruments. Each patient group was compared regarding changes in outcome measures one year after surgery. Between-group comparison was also performed.

**Results:**

Of the 458 patients with complete data, 15.3% and 9.6% had experienced presurgical anxiety and depression, respectively. All patient groups displayed statistical (*P* < 0.001) and clinical improvement in all outcome measures. Patients with presurgical anxiety and/or depression generally displayed less improvement, though the only significant mean differences concerned the Knee Injury and Osteoarthritis Outcome Score (KOOS)-sport score in the non-anxiety and non-depression groups (*P *= 0.006 and 0.03, respectively), a higher proportion of clinically improved KOOS pain in the non-anxiety group (*P* = 0.03), and the general health state in the anxiety and depression groups (*P *= 0.004 and 0.04, respectively).

**Conclusions:**

All patients improved in outcome measures 1 year after TKA, regardless of presurgical psychological state. Patients with presurgical anxiety and/or depression benefit greatly from surgery and should not be discriminated against based on presurgical psychological distress, though this fact should not eliminate the preoperative psychological assessment of patients.

**Level of evidence:**

II.

## Introduction

Total knee arthroplasty (TKA) has proven to be successful in improving pain and function among patients with knee arthritis [1–5]. Unfortunately, there are still patients who are not satisfied after TKA [6–10]. The number of such dissatisfied patients is expected to increase because of an increasing number of patients undergoing TKA [1, 11, 12]. Dissatisfaction rates reported in the literature range between 6% and 32%, and have been attributed to mechanical, psychological, and combined factors [10, 13–18].

Anxiety is defined as a disorder of persistent and exaggerated feelings of fear in relation to normal situations [19, 20]. The prevalence of anxiety in the general population as reported in the literature varies, and can be as high as 33% [19, 21]. Depression is a mental disorder that causes feelings of unhappiness and loss of pleasure in activities once enjoyed [22]. Only 4–8% of people have a clinical diagnosis of depression; in contrast, symptoms of depression are much more common, but only about one-third of patients with depression symptoms require treatment [23]. A study showed that approximately 20% of patients with osteoarthritis experience anxiety and/or depression symptoms [24].

Although patients’ expectations are the main factor contributing to patient satisfaction after TKA [6, 8, 16, 25–28], the role of psychological factors has been paid more attention in recent years [13, 14, 24, 26, 29–35]. One study found higher levels of 90-day readmission, dissatisfaction, and burden for healthcare providers among patients with depression [33]. Another study showed that patients with preoperative anxiety and pain catastrophizing had more postoperative pain, poorer preoperative and postoperative knee function, and a higher dissatisfaction rate; the authors recommended the preoperative screening and treatment of psychological factors [30].

One possible interpretation of the above-mentioned studies is that patients with anxiety and depression would not benefit from TKA surgery, and, hence, would be difficult for knee surgeons and other healthcare providers to handle postoperatively, given their repeated complaints of dissatisfaction. This might lead to the exclusion of patients who need to undergo TKA, potentially unnecessarily increasing patient suffering and paradoxically increasing the dissatisfaction rate. A global effort is now being made to prevent the increasing rate of dissatisfaction that might be expected due to the increasing number of TKA surgeries performed worldwide [1, 2, 11].

Despite the large number of studies investigating dissatisfied patients after TKA, little attention has been paid to the actual benefits of TKA surgery in patients with preoperative anxiety and depression. Our aim in this cohort study was, therefore, to investigate the prevalence of symptom improvement among patients with preoperative anxiety and/or depression in comparison with patients who did not have anxiety and/or depression. This might improve our understanding and evaluation of patients in whom knee complaints coexist with preoperative psychological distress; this might in turn decrease the unnecessary delay of TKA surgery.

## Method

### Patient selection

This was a prospective cohort study of a consecutive sample of Swedish patients who underwent TKA between April 2016 and July 2018. The patients received their primary total knee prosthesis at three hospitals in Mid-Sweden. All the orthopedic surgeons used a standard paramedian approach with one of three types of knee prosthesis: Genesis II (Smith & Nephew, Watford, UK), NexGen MBT (Zimmer-Biomet, Warsaw, IN, USA), and Journey TKA (Smith & Nephew, London, UK).

Inclusion criterion: all patients with knee arthritis (for primary osteoarthritis, secondary osteoarthritis, and inflammatory arthritis) who were scheduled for primary TKA.

Exclusion criteria: any patients having revision arthroplasty, medial unicompartmental arthroplasty, lateral unicompartmental arthroplasty, and patella-femoral arthroplasty.

All patients were asked to complete a questionnaire including patient-reported outcome measures (PROMs), regardless of whether or not they were included in the study. PROMs data are routinely collected before and one year after TKA for the Swedish Knee Arthroplasty Registry (SKAR) at the clinics where the study was conducted. These data were expected to cover around 500 patients during the study period (2016–2018), which was considered a representative sample. It was decided to include the first 500 consecutive patients with complete data.

SKAR was set up in 1975 and is now one of the most trusted such registries in the world [12]. It uses the individual-based registration of patients and TKA surgery. SKAR provided us with the PROMs questionnaire used annually by the registry, created software for entering and calculating the HADS score, and gave us access to its data program for entering PROMs, including HADS data. This access concerned only our patients and was restricted to the common database. We, therefore, took advantage of SKAR routines in terms of using a validated questionnaire and high-quality software that protects patient identity.

Demographic data for the patients included in the study are given in Table 1.

**Table 1:** Demographics of the TKA patients included in the study (*n* = 458)

Variables	PA (*n* = 70)	NPA (*n* = 388)	*P*-value	PD (*n* = 44)	NPD (*n* = 414)	*P*-value*
Gender, *n* (%)						
Female	43 (61)	196 (51)	0.09	29 (66)	210 (51)	0.05
Male	27 (39)	192 (49)		15 (34)	204 (49)	
Age, *m*	68	70	0.01	66	70	0.003
BMI	30	29	0.08	31	29	0.001
ASA class						
I	18 (26)	99 (25)	0.94	15 (34)	102 (25)	0.27
II	45 (64)	255 (66)		24 (55)	276 (66)	
III	7 (10)	34 (9)		5 (11)	36 (9)	
Charnley class						
A	14 (20)	98 (25)	0.37	7 (16)	105 (25)	0.21
B1	16 (23)	97 (25)		8 (18)	105 (25)	
B2	7 (10)	58 (15)		6 (14)	59 (14)	
C	32 (46)	131 (34)		22 (50)	141 (34)	
Missing	1 (1)	4(1)		1 (2)	4 (1)	
Diagnosis, *n* (%)						
Osteoarthrosis	65 (93)	377 (97)	0.29	42 (96)	400 (97)	0.61
Osteonecrosis	2 (3)	5 (1)		1 (2)	6 (1)	
Rheumatoid arthritis	2 (3)	3 (1)		0 (0)	5 (1)	
Fracture sequelae	1(1)	3(1)		1(2)	3 (1)	
OARSI-Responder, *n* (%)						
Non-responder	12 (17)	38 (10)	0.15	4 (9)	46 (11)	0.39
Responder	58 (83)	346 (90)		40 (91)	364 (89)	
Patella component	0 (0)	9 (2)	0.19	1 (2)	8 (2)	0.87
Previous surgery						
No	57 (81)	302 (78)	0.34	34 (77)	325 (80)	0.63
Yes	13 (19)	86 (22)		10 (23)	89 (20)	
Anesthesia, *n* (%)						
General	40 (57)	221 (57)	0.97	21 (48)	240 (58)	0.19
Spinal	30 (43)	167 (43)		23 (52)	174 (42)	
LIA, *n* (%)						
No	1 (1)	5 (2)	0.83	1 (2)	407 (98)	0.75
Yes	69 (99)	388 (98)		43 (98)	7 (2)	
Tourniquet, *n* (%)						
No	41 (59)	210 (54)	0.49	19 (43)	232 (56)	0.10
Yes	29 (41)	178 (46)		25 (57)	182 (44)	
Surgical time, min	89	87	0.51	85	88	0.33
Side, *n* (%)						
Right knee	42 (60)	193 (50)	0.11	26 (59)	209 (51)	0.27
Left knee	28 (40)	195 (50)		18 (41)	205 (49)	

*BMI* body mass index, *PA* preoperative anxiety, *NPA* no preoperative anxiety, *PD* preoperative depression, *NPD* no preoperative depression, *n* number,  *% *percent, *m* mean, *ASA* American Society of Anesthesiologists, *OARSI* Osteoarthritis Research Society International, *LIA* local infiltration anesthesia

*Chi-square test for binary data and independent t-test for continuous data

### Outcome measures

A psychometrically validated questionnaire was used to collect the data [36–38]. It was completed by the patients at two time points: before surgery and one year afterwards. This questionnaire included four different measures of PROMs: the Knee Injury and Osteoarthritis Outcome Score (KOOS), which consists of five subscales; general health state rated on a scale of 0–100; a visual analog scale (VAS) for rating pain intensity from 0 to 100; and a five-dimensional general quality of life instrument (EQ-5D-3L).

The questionnaire also included the Hospital Anxiety and Depression Scale (HADS), which consists of seven items for anxiety and seven items for depression. Each item has four possible answers, i.e., 0–3. HADS has a minimum score of 0 and a maximum of 21 for each dimension. HADS is a screening instrument designed mainly to explore anxiety and depression in patients with somatic and psychotic disorders [39]. It takes 2–5 min to complete and has been translated into many languages [32, 40]. It has good validity in defining anxiety and depression and in assessing their severity. It has been shown to be sensitive to change both during the disease course and in response to interventions [38–40].

We first categorized the patients, based on their HADS scores, into two groups: one with anxiety and another without anxiety; similarly, we obtained two groups with and without depression symptoms. Patients were regarded as having anxiety and/or depression symptoms if their relevant HADS score was more than seven [38].

Preoperative VAS expectations were assessed on a 0–100 scale, with 0 representing no pain and 100 the worst experienced pain. Postoperative VAS satisfaction was assessed using a similar 0–100 scale; the scale was completed by the patients one year after TKA and indicated the degree of satisfaction with their knee pain. “How good is your knee?” was another 0–100 scale (best–worst) used to assess postoperative patient satisfaction after one year [12].

### Analysis

Chi-square testing was used to compare binary data, while independent *t*-testing was used to compare continuous data for demographic variables, expectations, and satisfaction (Tables 1, 2, and 7).

**Table 2:** Demographics of the patients with incomplete data compared with complete data

Characteristics	Incomplete data, *n* (total *n* = 157)	Complete data, *n* (total = 458)	*P*-value*
Gender, *n* (%)			0.07
Female	95 (60)	239 (52)	
Male	62 (40)	219 (48)	
Age, *m*	68	70	0.06
BMI, *m*	30	29	0.003
ASA, *n* (%)			0.07
I	26 (16)	117 (25)	
II	115 (73)	300 (66)	
III	16 (10)	41 (9)	
Charnley class, *n* (%)			< 0.001
A	15 (9)	112 (25)	
B1	17 (11)	113 (25)	
B2	17 (11)	65 (14)	
C	53 (34)	163 (35)	
Missing	55 (35)	5 (1)	
PAS, *n* (%)			0.004
No	68 (72)	388 (85)	
Yes	26 (27)	70 (15)	
Missing	63		
PDS, *n* (%)	81 (87)	414 (90)	0.33
No			
Yes	12 (13)	44 (10)	
Missing	64		

*BMI* body mass index, *ASA* American Society of Anesthesiologists, *PAS* preoperative anxiety symptoms, *PDS* preoperative depression symptoms

*Chi-square test for binary data and independent t-test for continuous data

A paired *t* test was used to compare changes in mean differences in every group before surgery and one year after surgery regarding the outcome measures (i.e., EQ-5D-3L, VAS pain, general health state, and the KOOS subscales). We divided the patients into four groups (i.e., with preoperative anxiety, without preoperative anxiety, with preoperative depression, and without preoperative depression) and used a paired Student’s *t*-test to compare the mean differences in outcome measures in all four groups before and one year after TKA.

Two-way repeated-measures ANOVA was used to compare the mean differences within each group (i.e., anxiety, no anxiety, depression, and no depression) and to compare the mean differences between groups (i.e., anxiety vs. no anxiety and depression vs. no depression) (Tables 3 and 4).

**Table 3:** Change in outcome variables one year after TKA in patient groups without and with preoperative anxiety symptom

Outcome variable	No preoperative anxiety (*n* = 388)		Preoperative anxiety (*n* = 70)		MD between groups	Sig.^**^
	MD	Sig (2-tailed)^*^	MD	Sig (2-tailed)		
EQ-5D-3L	0.32	< 0.001	0.33	< 0.001	0.01	0.89
VAS pain	− 48	< 0.001	− 45	< 0.001	3	0.35
General health state	11	< 0.001	16	< 0.001	5	0.17
KOOS-symptoms	30.6	< 0.001	30	< 0.001	0.6	0.83
KOOS-pain	42	< 0.001	37.2	< 0.001	4.8	0.08
KOOS-ADL	33	< 0.001	30	< 0.001	3	0.18
KOOS-sport	28	< 0.001	18	< 0.001	9	0.006
KOOS-QoL	43	< 0.001	38	< 0.001	5	0.08

*MD* mean difference, *VAS* visual analog scale, *KOOS* Knee Injury and Osteoarthritis Outcome Score, *ADL* activities of daily living, *QoL* quality of life

*Paired *t*-test; ** Two-way repeated-measures ANOVA

**Table 4:** Change in outcome variables one year after TKA in patient groups without and with preoperative depression symptoms

Outcome variable	No preoperative depression (*n* = 414)		Preoperative depression (*n* = 44)			Sig.**
	MD	Sig (2-tailed)*	MD	Sig (2-tailed)	MD between groups	
EQ-5D-3L	0.32	< 0.001	0.33	< 0.001	0.01	0.86
VAS pain	− 48	< 0.001	− 49	< 0.001	1	0.78
General health state	11.5	< 0.001	14	< 0.001	2.5	0.54
KOOS-symptoms	30.5	< 0.001	31	< 0.001	0.5	0.89
KOOS-pain	41	< 0.001	40	< 0.001	1	0.80
KOOS-ADL	33	< 0.001	30	< 0.001	3	0.40
KOOS-sport	27	< 0.001	18	< 0.001	9	0.03
KOOS-QoL	43	< 0.001	37	< 0.001	6	0.15

*MD* mean difference, *VAS* visual analog scale, *KOOS* Knee Injury and Osteoarthritis Outcome Score, *ADL* activities of daily living, *QoL* quality of life

*Paired *t*-test; ** Two-way repeated-measures ANOVA

A Chi-square test was used to compare the proportions of patients with and without preoperative anxiety in relation to clinical improvement in EQ-5D-3L, VAS pain, general health state, and each KOOS subscale. A *P*-value of less than 0.05 was regarded as statistically significant. To determine whether the improvement of an outcome measure was clinically significant, specific cut-off points were used as follows: 0.25 for EQ-5D-3L, 15 for VAS pain, 15 for the general health state variable, and eight for each KOOS subscale [12, 36]. Similar comparisons were performed for patients with and without preoperative depression symptoms. Thus, cross tables were used to compare two categories of preoperative psychological state (i.e., with anxiety/depression and without anxiety/depression) in relation to the two categories of 1-year postoperative outcome measures (i.e., clinically significant improvement and non-clinically significant improvement) (Tables 5 and 6).

**Table 5:** Comparison between patient groups with and without preoperative anxiety symptoms in relation to postoperative improvement in outcome measures

Variable	PA *n* (%)	NPA *n* (%)	OR	*P*-value^*^
EQ-5D-3L				
CSI	37 (53)	222 (58)	1.08	0.46
No CSI	33 (47)	164 (42)		
VAS pain				
CSI	68 (97)	374 (98)	0.82	0.80
No CSI	2 (3)	9 (2)		
General health state				
CSI	38 (55)	138 (38)	1.41	0.004
No CSI	31 (45)	240 (62)		
KOOS-symptoms				
CSI	56 (80)	329 (85)	1.06	0.31
No CSI	14 (20)	59 (15)		
KOOS-pain				
CSI	62 (89)	365 (95)	1.07	0.03
No CSI	8 (11)	19 (5)		
KOOS-ADL				
CSI	59(84)	351 (91)	1.07	0.08
No CSI	11 (16)	35 (9)		
KOOS-sport				
CSI	43 (63)	267 (72)	1.14	0.12
No CSI	25 (37)	102 (28)		
KOOS-QoL				
CSI	65 (93)	355 (92)	0.99	0.85
No CSI	5 (7)	30 (8)		

*PA* preoperative anxiety, *NPA* no preoperative anxiety, *n (%)* number (percent), *OR* odds ratio, *P*-*value* probability value, *CSI* clinically significant improvement

*Chi-square test

**Table 6:** Comparison between patient groups with and without preoperative depression symptom in relation to postoperative improvement in outcome measures

Variable	PD *n* (%)	NPD *n* (%)	OR	*P*-value*
EQ-5D-3L				
CSI	22 (51)	237 (57)	1.12	0.43
No CSI	21 (49)	176 (43)		
VAS pain				
CSI	41 (95)	401 (98)	1.02	0.32
No CSI	2 (5)	9 (2)		
General health state				
CSI	23 (52)	153 (38)	1.33	0.04
No CSI	21 (48)	250 (62)		
KOOS-symptoms				
CSI	41 (93)	386 (94)	1.01	0.79
No CSI	3 (7)	24 (6)		
KOOS-pain				
CSI	42 (93)	386 (94)	1.01	0.79
No CSI	3 (7)	24 (6)		
KOOS-ADL				
CSI	39(89)	371 (90)	1.01	0.76
No CSI	5 (11)	41 (10)		
KOOS-sport				
CSI	30 (68)	280 (71)	1.04	0.67
No CSI	14 (32)	113(29)		
KOOS-QoL				
CSI	39 (91)	381 (93)	1.02	0.76
No CSI	4 (9)	31 (7)		

*PD* preoperative depression, *NPA* no preoperative depression, *n* (%) number (percent), *OR* odds ratio, *P*-value probability value, *CSI* clinically significant improvement

*Chi-square test

VAS expectations were sorted into three categories: high expectations (0–30), moderate expectations (30–60), and low expectations (60–100). Similar categories were formed for VAS satisfaction, i.e., high/very high satisfaction, moderate satisfaction, and low/very low satisfaction. Similarly, we categorized the “How good is your knee?” variable as good/very good knee, moderately good knee, and bad/very bad knee. The mean values for VAS expectations, VAS satisfaction, and “How good is your knee?” were also calculated (Table 7) [12]. SPSS, version 25 (IBM, SPSS Inc., Chicago, Illinois, USA) was used for the data analysis.

**Table 7:** Patients’ preoperative expectations and postoperative satisfaction (*n* = 458)

Variables	PA (*n* = 70)	NPA (*n* = 388)	*P*-value*	PD (*n* = 44)	NPD (*n* = 414)	*P*-value*
VAS expectations, *n* (%)						
High expectations	66 (92)	455 (92)	0.99	41 (93)	379 (92)	0.71
Moderate expectations	4 (7)	28 (7)		3 (7)	29 (7)	
Low expectations	1 (1)	5 (1)		0 (0)	6 (1)	
VAS expectations, *m*	18.45	16.65	0.28	16.13	17	0.66
VAS satisfaction, *n* (%)						
High/very high satisfaction	56 (78)	315 (81)	0.41	35 (80)	337 (80)	0.54
Moderate satisfaction	5 (8)	37 (10)		3 (7)	40 (10)	
Low/very low satisfaction	9 (13)	32 (9)		6 (13)	37 (9)	
VAS satisfaction, m	22	17	0.12	21	17	0.30
“How good is your knee?”, *n* (%)						
Good/very good knee	46 (65)	274 (70)	0.62	28 (64)	292 (70)	0.73
Moderately good knee	17 (25)	87 (22)		12 (27)	92(22)	
Bad/very bad knee	7 (10)	27 (7)		4 (9)	30 (7)	
“How good is your knee?”, m	29	23	0.03	28.53	23.68	0.18

*VAS* visual analog scale, *PA* preoperative anxiety, *NPA* no preoperative anxiety, *PD* preoperative depression, *NPD* no preoperative depression, *n* number, %  percent, *m* mean

*Chi-square test for binary data and independent *t*-test for continuous data

## Results

**Fig. 1:**
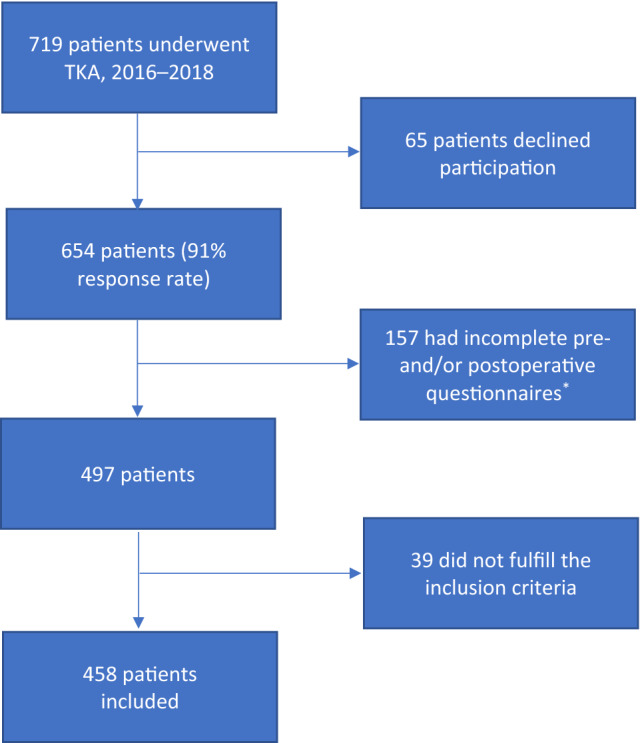
Flow chart of the patients included in the study. *Reasons for missing data were: (1)At the beginning of the study the nurses had not yet got into the routine of distributing the questionnaire, which caused them to miss some patients; (2)Patients delaying their answers for more than 3 months; (3) Incomplete answering of the questionnaire; (4) Patients not returning the preoperative or postoperative questionnaire despite reminders; (5) Death

Between April 2016 and July 2018, 719 patients from Mid-Sweden underwent knee arthroplasty. The participation response rate was 91% (65 patients declined). After excluding patients who declined (*n *= 65), those with incomplete data (*n* = 157), and those who did not meet the inclusion criteria (*n* = 39), a total of 458 patients with complete data were included in the study (Fig. 1).

Missing data were distributed randomly and did not affect any specific group or hospital. The demographics of the patients with missing data are shown in Table 2. Patients with incomplete data had significantly higher body mass index (BMI; *P* = 0.003), a lower percentage of Charnley class A and B (*P* < 0.001), and probably more preoperative anxiety (*P* = 0.004). There were no statistically significant differences in other variables, as shown in Table 2.

We first categorized the data according to the preoperative anxiety and depression state: 70 patients (15.3%) had preoperative anxiety symptoms and the remaining 388 (84.7%) did not have anxiety, while 44 patients (9.6%) had preoperative depression symptoms and the remaining 414 (90.4%) did not have depression.

Demographic data analysis revealed no statistically significant differences between the groups with and without preoperative anxiety and/or depression (Table 1), except that patients with preoperative anxiety and/or depression were younger than those without anxiety and/or depression (*P* = 0.01 and 0.003, respectively). The mean BMI in the group with preoperative depression was significantly higher than in the group without preoperative depression (31 vs. 29, *P* = 0.001).

Comparisons were performed for each group before and one year after surgery. All groups displayed statistically and clinically significant improvement in all outcome measures (Tables 3 and 4): EQ-5D-3L, VAS pain, general health status, and all KOOS subscales (i.e., symptoms, pain, activities of daily living, sport, and quality of life; *P* < 0.001).

Patients with preoperative anxiety and/or depression generally displayed less improvement in the outcome measures than did the groups without anxiety and/or depression, but most of the differences were not statistically significant. One exception was the mean difference in KOOS-sport, which was significantly higher in the non-anxiety and non-depression groups than in the anxiety and depression groups; moreover, two of the further three significant results were in favor of the anxiety and/or depression groups (Tables 5 and 6). The only statistically significant differences were in clinically significant improvements of KOOS-pain in 95% of patients without preoperative anxiety versus 89% of patients with preoperative anxiety (*P* = 0.03), of the general health state in 55% of patients with preoperative anxiety versus 38% of patients without preoperative anxiety (*P* = 0.004), and of general health state in 52% of patients with preoperative depression versus 38% of patients without preoperative depression (*P* = 0.04) (Tables 5 and 6).

Our data indicated that 92% of patients had high preoperative VAS expectations, 81% high/very high VAS satisfaction, and 70% good/very good knee one year after TKA. Patients without preoperative anxiety and/or depression had slightly higher values for the preoperative VAS expectations, VAS satisfaction, and “How good is your knee?” variables. The differences among either the proportions or mean values were not statistically significant, however, except in the “How good is your knee?” variable, for which the mean value in the non-anxiety group was statistically significantly higher than in the anxiety group (23 vs. 29, *P* = 0.03) (Table 7).

## Discussion

This study examined clinical improvements one year after total knee arthroplasty in patients with knee arthritis. The results indicate significant improvements in outcome measures regardless of the patients’ presurgical anxiety and/or depression symptoms. Although patients with presurgical anxiety and/or depression symptoms generally displayed less improvement than did patients without these symptoms, the differences between the groups were not statistically significant in this cohort except in the case of pain and sport/recreational activities.

Many studies have shown that TKA is an effective procedure for improving pain and function in patients with knee arthritis [1, 4, 15, 16]. However, there is an ongoing worldwide effort to further improve the outcome after TKA, because of the expected increase in the number of patients undergoing TKA, which entails a risk of increasing the dissatisfaction rate after surgery [1]. Our study is in agreement with the literature regarding the improvement of both generic and knee-specific outcome measures one year after TKA. The highest improvement was seen in VAS pain and KOOS-QoL (i.e., quality of life), regardless of presurgical psychological state.

In the past 7 years, more attention has been paid to the negative effects of presurgical psychological distress on patient satisfaction after TKA [13, 14, 29–32], leading to the improved presurgical assessment of patients and improved patient selection for surgery. One study revealed preoperative anxiety and/or depression to be an important factor predicting patient satisfaction after TKA [14]. The authors highlighted that patients with preoperative anxiety and/or depression had six times more risk of being dissatisfied than did patients without these symptoms; this study and other studies recommended preoperative psychological assessment to possibly decrease the dissatisfaction rate [14]. Another systemic review also focused on preoperative psychological distress (i.e., psychological anxiety and/or depression) as a predictor of poor outcome after TKA [13].

Many of the above-mentioned studies strove to identify patients with psychological illness, which might be misinterpreted by orthopedic surgeons as indicating that they should not operate on patients with preoperative psychological illness. This delay of surgery in patients with presurgical psychological distress has not yet been well studied in the literature, but it is not uncommon in clinical practice. Our study, however, highlights that TKA is also effective in patients with preoperative anxiety and/or depression symptoms, as these patients experienced statistically and clinically significant improvement in both generic and knee-specific outcome measures. The present results can help to increase awareness and accurate assessment of this category of patients, which might in turn prevent unnecessary delays of surgery and hence improve these patients’ quality of life.

Although patients with presurgical anxiety and/or depression displayed less improvement than did patients without such symptoms, the within-group improvement was still considered large and both clinically and statistically significant. Moreover, the mean differences between the groups with and without anxiety and/or depression symptoms were not statistically significant, except in the case of sport and recreational activities. Further studies with larger sample sizes might be able to uncover statistically significant differences between the groups, though these will not necessarily be clinically important. Another exception to the above was that a significantly higher proportion of patients without presurgical anxiety saw an improvement in KOOS-pain than did those with presurgical anxiety symptoms.

Another interesting result concerned the patients’ general health state: a significantly higher proportion of patients with preoperative anxiety and/or depression displayed improvement in this measure than did those without such symptoms. This might offer further evidence of the effectiveness of TKA in improving general quality of life, reducing pain, and improving knee function in patients with preoperative anxiety and/or depression symptoms. The SKAR annual report showed an improvement in the general health state of TKA patients one year after the performance of surgery [12], though there was no stratification of the groups based on patients psychological distress (anxiety/depression symptoms).

Patients’ preoperative expectations in relation to postoperative satisfaction have also been studied [6, 8, 25–28], showing that fulfillment of patients’ expectations predicted postoperative patient satisfaction after TKA surgery. In a qualitative study, we found that patients’ expectations were still the major contributing factor affecting patient contentment one year after surgery [25].

In this study, the differences between patients with and without preoperative anxiety and/or depression symptoms were not statistically significant. These results provide further evidence that patients with preoperative anxiety and/or depression symptoms have as high preoperative expectations and postoperative satisfaction as do patients without these symptoms. Increasing the size of the patient sample in future studies might reveal statistically significant differences. These differences should not lead to discrimination against patients at a group level, but rather should help TKA surgeons improve their assessment of patients at the individual level.

## Strengths and weaknesses

**Table:** 

*n*	Limitations	*n*	Strength
1	Insufficient data for between-group comparison	1	Sufficient data for within-group comparison
2	No objective physical knee tests	2	Consecutive prospective sample

We obtained complete data from 458 patients, which was sufficient to yield clinically and statistically significant results regarding within-group comparisons. However, this patient sample was insufficient to yield statistically significant results regarding the comparison of patients with versus without anxiety and/or depression in relation to clinical improvement in the KOOS subscales.

Another weakness of this study was that improvement was considered only on the basis of PROMs. We did not administer any objective physical tests such as walking tests or stair-climbing tests, and we did not directly ask the patients whether they were really satisfied with the surgery one year postoperatively.

A strength of this study was its prospective cohort design, including a consecutive group of knee arthritis patients who underwent surgery during 2016–2018. This helped minimize the selection bias, even though 24% (157/654) of cases were lost.

## Conclusions

TKA produces excellent subjective improvement in patients’ quality of life, pain, activities of daily living, knee symptoms, recreational activities, and general health state. Patients with presurgical anxiety and/or depression symptoms should be accurately assessed before surgery; however, they benefit greatly from surgery and should not be discriminated against based on presurgical psychological distress.
